# Effects of Early-Stage Treeline Shifts on Soil Microbial Biomass and Catabolic Diversity in Reserved and Grazed Subalpine Meadows

**DOI:** 10.3390/plants14101541

**Published:** 2025-05-20

**Authors:** Kristina Ivashchenko, Anastasiya Romanova, Sofia Sushko, Anna Zhuravleva, Anna Kvitkina, Anna Khodzhaeva, Nadezhda Ananyeva

**Affiliations:** Institute of Physicochemical and Biological Problems in Soil Science, Russian Academy of Sciences, Pushchino 142290, Moscow Region, Russia

**Keywords:** carbon, nitrogen, phosphorus, soil microbial diversity, incubation experiment, plant residue quality

## Abstract

Treelines are advancing upward on mountain slopes due to climate warming and reduced grazing intensity. However, the effects of initial vegetation changes on soil C, N, and P retention, microbial biomass, and catabolic diversity in the subalpine meadows during the early stages of treeline shifts remain poorly understood. This research aimed to better understand the direction and drivers of microbial processes related to C, N, and P cycles in the soil of subalpine natural and grazed meadows, with treatments involving meadow grasses alone (GR, control) and as a mixture with forest litter, specifically birch leaves (BLs), in a one-year microcosm experiment. The addition of BLs with GR resulted in a 12–67% decrease in the retention of C, N, and P in soil microbial biomass, but an 8–9% increase in catabolic diversity compared to the control. The most pronounced effect was observed in the N content of the soil microbial biomass (MBN) for both land uses. The increased proportion of recalcitrant plant residue fractions (acid-insoluble and non-polar extractables) contributed to the decrease in soil MBN content. This shift also reduced the microbial metabolic response to carbohydrates in total substrate-induced respiration, leading to a more balanced and catabolically diverse microbial community. These results improve our understanding of the early response of C, N, and P cycling in mountain soils to treeline shifts mediated by climate warming.

## 1. Introduction

Rising air temperatures promote upward treeline migration on mountain slopes [1]. Recently, treeline shifts have been observed in many mountainous areas of the world and have been linked to global climate warming [2,3,4,5]. Anthropogenic land use of mountain lands is another well-known driver of treeline dynamics [6]. Highly productive subalpine meadows in the Alps, Caucasus, Pyrenees, Carpathians, and other mountain systems have historically served as pasturelands. Intensive grazing pressure usually inhibits tree regeneration and limits the expansion of the forest into subalpine meadows. In addition, a reduction in the intensity of grazing or its complete cessation stimulates this expansion [6]. It has been demonstrated that grazing limits the regeneration of both *Pinus brutia* and *Cupressus sempervirens* [7]. Notably, this study also revealed that decreased soil trampling pressure from grazing allowed the natural regeneration of woody species. Therefore, the history of land use should be taken into account when studying the consequences of the shift from mountain forest belts to meadows.

Climate warming, coupled with reduced grazing intensity, is changing the vegetation of mountain meadows through the encroachment of woody plants and the replacement of ground cover grasses. This could lead to an increase in the proportion of recalcitrant compounds in plant residues and a decrease in the proportion of labile compounds. Our previous research has shown that the levels of complex C-aromatic compounds in plant residues are increased in mountain forests compared to subalpine meadows [8]. As a result, the chemical composition of soil organic matter (SOM) will change, affecting its turnover rate [9]. It has been suggested that labile plant substrates contribute to carbon (C) retention in the soil because microorganisms use them more efficiently, producing organic compounds that are stabilized by binding to the mineral matrix [10]. On this basis, it can be assumed that the long-term development of forests on subalpine meadows will lead to a decrease in soil C retention due to the input of more complex plant material into the soil. At the same time, it is possible that the opposite effect will occur in the short term when the diverse plant material is introduced into the soil. This has been demonstrated for agricultural soils. Specifically, compared to the addition of labile cellulose-rich material alone, a mixture of recalcitrant and labile plant residues resulted in an increase in C retention in the microbial biomass during the 56 days of its decomposition [11]. It has also been shown that plant residue diversity leads to a more diverse soil microbial community [12,13], which could contribute to the SOM turnover rate.

In general, for mountain soils, changes in soil C, nitrogen (N), microbial biomass content, and microbial activity have been evaluated along the altitudinal gradient to predict the consequences of upward vegetation shifts [14,15,16]. However, due to the complex interactions between abiotic and biotic factors, often fail to clearly reveal the mechanisms of changes in vegetation cover and its quality on mountain soils in altitudinal studies. Therefore, microcosm experiments are needed to identify changes in processes related to C, N, and even phosphorus (P) cycling to predict the early soil consequences of vegetation quality changes under treeline shifts.

In microcosm experiments, plant residues from different plant parts (roots, leaves, twigs, etc.) or plant species are commonly used to study microbial processes related to SOM turnover [11,17,18,19,20]. The quality of plant residues is usually characterized by C/N content, N content, or the lignin/N ratio [10,11,21]. For example, a low C/N ratio in plant residues contributes to enhanced microbial decomposition in the soil, releasing more N compared to a high C/N ratio [10,22]. However, the effect of plant residues on decomposition dynamics and the fate of C, N, and P in soils may be complex and cannot be fully explained by the stoichiometry of the elements in plants. As an alternative, it would be useful to consider the composition of plant residues, such as the proportion of non-polar extractives, water-soluble fractions, and acid-soluble and acid-insoluble fractions. These fractions may directly affect the structure of soil decomposers and the rate of microbial processes, but this effect is poorly understood in the scientific literature.

Thus, a critical knowledge gap remains regarding the short-term effects of changes in vegetation composition on C, N, and P retention in soil microbial biomass and catabolic diversity during treeline shifts. Our research aimed to better understand the specific mechanisms driving the dynamics of microbial processes in the soil of subalpine natural and grazed meadows during the early stages of plant material mixing, such as forest litter and meadow grasses. A one-year microcosm experiment was conducted for this purpose. We propose the following hypotheses:

**H1:**  *A shift in the treeline during the early successional stage leads to a decrease in C, N, and P retention as soil microbial biomass due to an increase in the proportion of recalcitrant organic compounds introduced with forest litter.*

**H2:**  *The addition of forest litter to grass residues contributes to a more diverse and complex material, providing a high level of catabolic diversity of soil microorganisms under the shifting treeline.*

## 2. Results

### 2.1. Plant Residues

**Figure 1:**
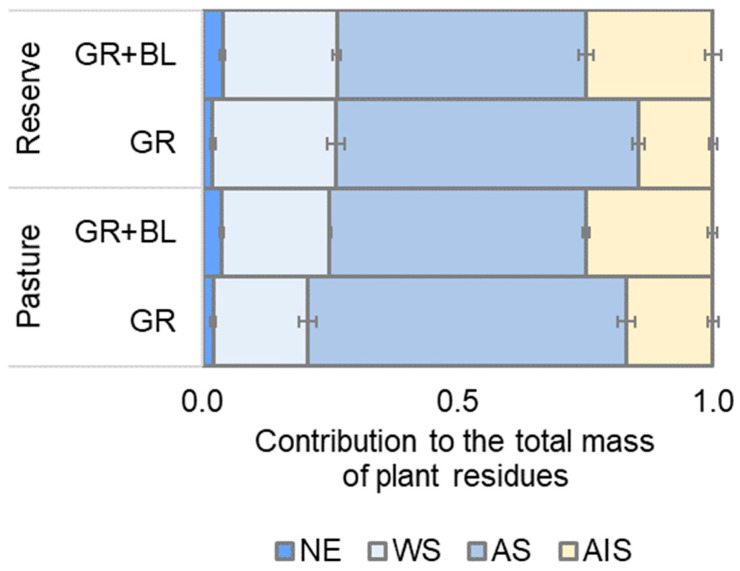
The non-polar extractive (NE), water-soluble (WS), acid-soluble (AS), and acid-insoluble (AIS) fractions of the plant residues used in the experiment: subalpine grass (GR) and a mixture of GR and birch leaves (GR + BLs) collected from reserved and grazed mountain slopes. Mean ± standard error for *n* = 3.

At the end of the trial, most of the plant residues had decomposed and remained at 14–17% of their initial weight. The plant residues added to the soil in the 1-year trial were of different qualities according to the experimental design (Figure 1). The addition of a mixture of birch leaves (BLs) and subalpine GR residues leads to a complexity of the plant material for microbial decomposition. This is demonstrated by a 1.5- to 1.7-fold increase in the acid-insoluble (AIS) fraction (e.g., lignin) and a 1.8- to 2.0-fold increase in the non-polar extractable (NE) fraction. The proportion of the water-soluble (WS) fraction did not change significantly, but the proportion of the acid-soluble (AS) fraction decreased by 1.2-fold in a mixture of BLs and subalpine GR residues compared to GR residues.

According to the C:N ratio in plant residues (above-ground GR biomass and forest litter), our previous studies have shown a decreasing trend from meadow to forest [8]. Thus, the C:N ratio does not always reflect the chemical complexity of plant residues.

### 2.2. Soil C, N, and P Content

**Figure 2:**
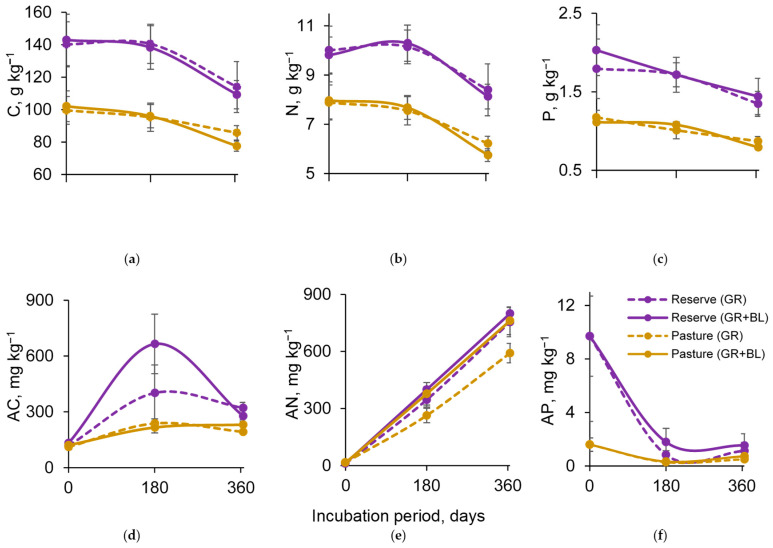
Dynamics of total carbon (**a**), nitrogen (**b**), and phosphorus (**c**) contents and their available forms (**d**–**f**) in soils of subalpine reserved and grazed meadows under the mineralization of grass (GR) and mixed residues of GR and birch leaves (GR + BLs). Mean ± standard error for *n* = 3.

Total soil C, N, and P, as well as available C (AC) and available P (AP), were higher in the reserved subalpine meadow than in the grazed meadow (Figure 2a–d,f). After 1 year of incubation of the soil samples with plant residues, the total soil C, N, and P and AP contents decreased for both land uses. At the end of the experiment, the soil AC increased by 2.1–2.7 times and 1.7–1.9 times for the reserve and the pasture, respectively (Figure 2d). However, the increase in the soil available N (AN) was characterized by a linear trend and was more pronounced (Figure 2e).

The effect of the quality of plant residues on soil C, N, and P was observed at the end of the experiment; however, for their available forms, it was already observed after 6 months of incubation (Figure 2).

At the end of the experiment, the decomposition of BLs with GR resulted in greater losses in soil CNP by 3–10% compared to the decomposition of GR alone. An exception was observed for the soil P content in reserved meadows, which showed an increasing trend. The addition of BLs increased AN and AP in the soil samples by 16–43% and 18–114%, respectively, compared to the samples with GR residue addition alone after 6 months of incubation. Interestingly, these changes were more pronounced for soil AN in grazed meadows, while soil AP showed a more pronounced increase in reserved meadows. The change in soil AC related to the quality of plant residues was not clearly established and depended on the incubation period and the land use type.

Thus, the shifting of the treeline in the early stages may accelerate the total losses of C, N, and P in meadow soils while enriching them with their available forms, regardless of the type of land use.

### 2.3. C, N, and P Content in Soil Microbial Biomass

**Figure 3:**
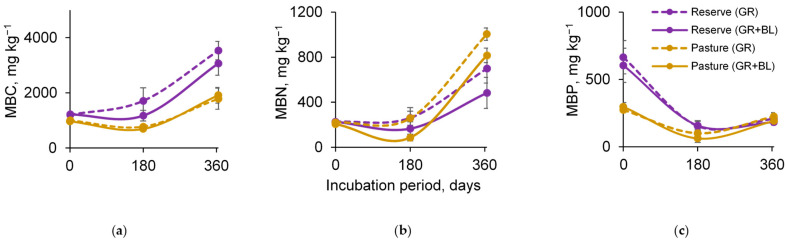
Dynamics of soil microbial biomass carbon (**a**), nitrogen (**b**), and phosphorus (**c**) contents for subalpine reserved and grazed meadows under mineralization of grass (GR) and mixed residues of GR and birch leaves (GR + BLs). Mean ± standard error for *n* = 3.

Consistent with the increases in available AC and AN, soil microbial biomass carbon (MBC) and nitrogen (MBN) increased during the decomposition of plant residues (Figure 3a,b). In contrast, microbial biomass phosphorus (MBP) gradually decreased toward the end of the experiment, mirroring the trend observed for AP (Figure 3c).

The addition of BLs to the reserved meadow soil resulted in a 13–31% reduction in MBC and a 31–37% reduction in MBN (Figure 3a,b; Table S1). The same trend was found for MBN in the grazed meadow soil (reduction of up to 19–67%). Plant residue quality had a lesser effect on soil MBP content, with a reduction trend observed in the reserved meadows (12%) after 1 year of decomposition and in grazed meadows (15–38%) for both incubation periods.

Thus, in the first stages, the treeline shift on subalpine meadows decreased the immobilization of C, N, and P in soil microbial biomass. The soil of the grazed meadow proved to be the most sensitive to the decrease in soil MBP, but the reserved meadow was characterized by a more pronounced change in soil MBC.

### 2.4. Catabolic Activity and Diversity of the Soil Microbial Community

**Figure 4:**
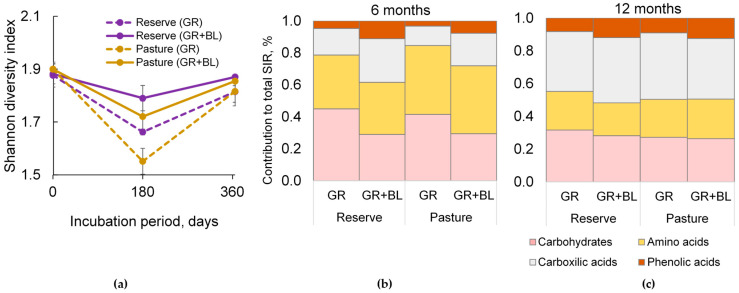
Shannon diversity index (**a**) and the averaged contribution of microbial respiration response to organic substrates in total substrate-induced respiration (SIR) after 6 (**b**) and 12 (**c**) months of decomposition of grass (GR) and mixed residues of GR and birch leaves (GR + BLs) in meadow soil samples collected from reserved and grazed mountain slopes. Mean ± standard error for *n* = 3.

The addition of BLs to the meadow soil increased microbial catabolic diversity by 8% for the reserve and 9% for the pasture after 6 months of incubation; however, the effect was smaller at the end of the experiment (Figure 4a). This change was accompanied by a decrease in the proportion of carbohydrate-consuming microorganisms and an increase in the proportion of microbial groups using both more complex phenolic compounds and more available carboxylic acids (Figure 4b). At the end of the experiment, the same trend in catabolic activity distribution was observed, but the effect was less pronounced than at mid-term (Figure 4c).

Regardless of land use, a treeline shift toward meadows is expected to increase soil microbial catabolic diversity and support a balanced respiratory response to different types of organic substrates.

### 2.5. Quality of Plant Residues and Dynamics of Soil Microbial Properties

The initial quality characteristics of plant residues (proportions of different organic fractions) had a greater influence on changes in microbial properties after 6 months of incubation of the soil samples than at the end of the incubation period (Table 1). The WS fraction explained 31–57% of the variation in soil MBC content, Shannon index (catabolic diversity), and relative microbial response to carboxylic acids and phenolic acids. These soil microbial properties increased with the proportion of the WS fraction (Figure S1). Soil catabolic diversity also increased with the NE fraction but decreased with the AS fraction. This may be because a higher NE fraction reduced the relative activity of microbial groups responding to carbohydrates while increasing the relative microbial response to carboxylic acids. Conversely, the AS fraction showed the opposite pattern, with a positive, strong relationship to the microbial response to carbohydrates and a negative relationship to the microbial response to carboxylic acids. Soil MBN was strongly influenced by the AIS fraction (explained variance is 43%) and, to a lesser extent, by the AS and NE fractions (26%). Increasing the AIS and NE fractions reduced N immobilization in microbial biomass, whereas an increase in the AS fraction showed the opposite trend. Soil MBP was not influenced by the quality characteristics of plant residues.

At the end of the incubation period, the quality of the plant residues played a significant role in the activity of the microbial group consuming amino acids (Figure S2). The highest contribution was observed for the AS fraction (explained variance is 46%), as its proportion increased with the relative microbial response to amino acids.

## 3. Discussion

### 3.1. Early Changes in Soil Microbial Biomass and Activity Induced by Plant Residue Quality

The short-term experiment with plant residues of different quality showed a decreasing trend in soil microbial biomass but an increasing functional diversity of the microbial community regardless of land use types (Figure 3 and Figure 4a). This finding aligns with in situ observations along meadow–forest transects in the study area in the northwestern Caucasus, which showed a decrease in soil MBC [15] but a significant increase in microbial functional diversity [23]. Differences in vegetation cover and associated residue quality explain the patterns observed. As the treeline shifts, the proportion of lignin components, fats, and waxes in plant residues is expected to increase (Figure 1). Together with the cellulose-containing GR litter, this will provide a wide range of organic compounds in the soil that influence the mineralization rate and the subsequent fate of C, N, and P in the soil [11,24,25]. In our study, the retention of C, N, and P elements in soil microbial biomass was reduced in samples with a mixture of BLs and GR residues compared to the addition of GR residues alone (Figure 3). According to the two-phase double exponential model [26], in the first stage, the natural polymers (cellulose, hemicellulose) decompose rapidly, stimulating C and N release [27]. Therefore, we observed significant increases in AC and AN after 6 months of soil incubation (Figure 3a,b), without substantial losses in their total content (Figure 2a,b). More recalcitrant organic compounds, such as lignin and waxes, are slowly degraded by soil microorganisms, and the process requires considerable energy to break the bonds [28,29,30]. Consequently, a higher lignin fraction contributes to increased microbial energy expenses, limits carbon acquisition, and reduces microbial efficiency [27,31]. This may be one of the reasons for the decrease in C, N, and P content in the microbial biomass during the decomposition of BLs in the meadow soil. Notably, the effect of residual quality was the most pronounced for soil MBN from both the reserved and the grazed mountain slopes (Figure 3b). The proportion of AIS, or the lignin-containing fraction in plant residues, contributed to the reduction in soil MBN content (Table 1). During the decomposition process, N compounds condense with polyphenols, the metabolites of lignin degradation, inhibiting soil microbial activity [26,29] and reducing the rate of N incorporation into microbial biomass. The inhibitory effect of the presence of lignin in soil samples was evident after 12 months of incubation. Specifically, the respiration response of the amino acid-utilizing microbial group decreased as the proportion of the AIS fraction increased (Table 1).

### 3.2. Enhancing Microbial Catabolic Diversity with Plant Residue Recalcitrance

Changes in microbial catabolic diversity following the input of plant residues into soil can be interpreted as a reflection of the degradation stages of different organic compounds, which differ in the energy required for their decomposition. During the first stage of decomposition, microbes actively synthesize C-acquiring enzymes, such as cellobiohydrolase, *β*-xylosidase, and *β*-glucosidase, which decay cellulose and hemicellulose to easily assimilable carbohydrates [32]. Concurrently, the demand for N required for microbial growth and enzyme synthesis increases, which subsequently induces the production of N-acquiring enzymes [31]. Likely for these reasons, after the first 6 months of plant residue decomposition, we observed a high imbalance in the microbial catabolic diversity directed toward the rapid utilization of simple carbohydrates and amino acids (Figure 4). Further, the higher the initial content of cellulose and hemicellulose (AS fraction) in plant residues is, the higher the microbial shift toward carbohydrate utilization and hence the imbalance in overall catabolic diversity (Table 1). Simultaneously, under conditions of an abundant source of available energy (i.e., carbohydrates), there is a parallel breakdown of more recalcitrant organic compounds, namely lignin [33,34]. In the early stage, the lignin degradation rate is positively correlated with its initial content in plant tissues [34]. This, in turn, may lead to a proportional enrichment of the soil with lignin degradation products, namely phenolic monomers. As a result, we observed a higher microbial respiratory response rate to phenolic acids in the soil amended with high-lignin residues (a mixture of GR and BLs), thus contributing to a more balanced catabolic diversity (Figure 4). However, as the mass loss of plant residues was greater than 80% (after 12 months), the effect of the initial residue composition on microbial catabolic diversity was markedly weakened (Table 1). This can be explained by changes in the chemical composition of the remaining plant matter during the decomposition process. As the cellulose breaks down during the rapid mass loss of plant residues, the relative amount of lignin, which decomposes more slowly, increases [26]. As a result, cellulolytic enzyme activity tends to decrease in the late stages of residue degradation, whereas ligninolytic enzyme activity tends to increase [31,32]. At this late stage, total catabolic activity was mainly determined by the initial lignin concentration in the plant tissues, as has been shown for leaf litter from 12 herbaceous species [35]. Here, more complex multistage biochemical processes of decomposition of recalcitrant organic compounds predominate, releasing a greater variety of organic molecules. Given the selective sorption of different organic molecules by minerals [36], the soil’s capacity to stabilize C increases with the chemical diversity of the plant residue inputs. Thus, increasing the chemical complexity of plant residues by shifting the treeline to subalpine meadows simultaneously increases microbial catabolic diversity and improves soil C stabilization in the mineral-associated fraction [8].

### 3.3. Study Limitations and Future Research

Our laboratory experiment revealed the short-term effects of changes in plant residue quality caused by the upward shift in the mountain treeline on microbially mediated C, N, and P cycling in meadow soils when other environmental factors are standardized (plant residue quantity, hydrothermal conditions) or excluded (e.g., root exudate inputs, rhizosphere microbial community). This study design has made it possible to disentangle the effect of residue quality, namely increased lignin content, from that of other factors. Regardless of the initial soil C, N, and P levels, similar trends in microbial biomass and catabolic diversity were observed in both reserved and grazed meadow soils with lignin-rich residue inputs. On the one hand, this may indicate stable mechanisms regulating soil microbial functioning and C, N, and P cycling associated with residue quality inputs. On the other hand, these results have limitations for extrapolation to field conditions, as they do not account for complex multifactorial effects, such as variations in residue quantity inputs, root exudate supply, the functioning of the rhizosphere microbial community, and fluctuating hydrothermal conditions. Future research is needed to verify these findings in situ. This can be achieved using a soil transplant experiment under field conditions, i.e., moving a meadow soil monolith to treeline conditions. Such an experiment, together with existing laboratory results, will allow us to fully assess the role of plant residue quality in the early restructuring of microbially mediated C, N, and P cycling in mountain soils.

## 4. Materials and Methods

### 4.1. Study Sites

**Figure 5:**
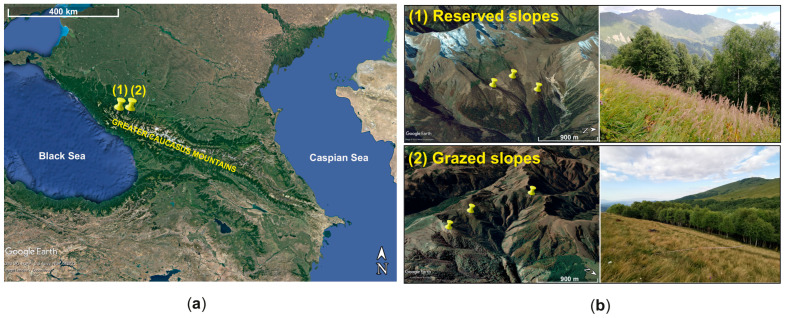
Scheme of location (**a**) and general view (**b**) with the study sites (yellow mark) in the reserved and grazed slopes of the northwestern Caucasus, Russia.

Subalpine meadows were selected from three reserved slopes at 2145–2235 m asl (43°43′ N; 40°43′ E) and three grazed slopes at 1882–1946 m asl (43°43′ N; 41°12′ E) in the northwestern Caucasus, Russia (Figure 5). The reserved slopes were located in the Caucasian State Nature Biosphere Reserve, established in 1924. The grazed slopes, located 40 km to the east of the reserve, have been used for a long time (over 100 years) for seasonal grazing by cattle, horses, and sheep.

The study area is part of the spur system of the Peredovoy Range of the Greater Caucasus. All slopes featured northeasterly facing aspects with an average slope of 27°. The climate of the study area is moderately continental, with mean annual air temperatures of 5.1–8.5 °C and annual precipitation of 1850 mm [8]. Soils were classified as Humic Cambisols with pH 4.7–4.8 [16]. Parent materials include metamorphic and sedimentary rocks, such as andalusite–mica schists, amphibolite depositions, and mudstones [37]. The vegetation cover is mainly represented by graminoids, including *Calamagrostis arundinacea* (L.) Roth, *Nardus stricta* L., and *Agrostis capillaris* L. The dominant species in the treeline was *Betula* sp., aged 45–60 and 18–22 years on reserved and grazed slopes, respectively.

### 4.2. Sampling and Treatments

**Figure 6:**
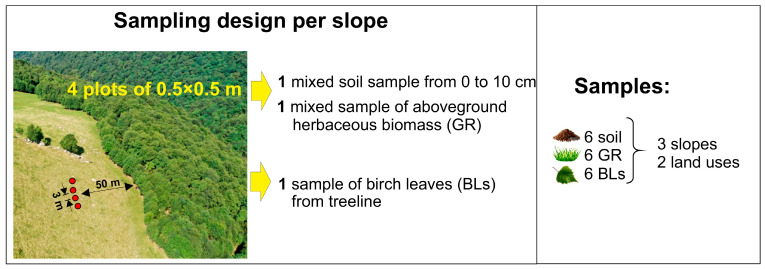
Design of soil and plant biomass sampling. Sampling sites are marked by red plots.

In early September 2022, we collected soil samples (0–10 cm depth) and aboveground biomass of herbaceous species (GR residue) from four 0.5 × 0.5 m plots spaced 3 m apart and located 50 m from the treeline within each slope (Figure 6). Soil samples were obtained for each plot after a geobotanical survey and cutting of aboveground biomass. The characteristics of the grass cover on the plots are given in Table 2. BLs were collected at the treeline close to sampling sites. For each slope, samples from the four plots were composited, yielding one composite soil sample, one GR residue sample, and one BL sample per slope (six slopes total). Soil samples were sieved (3 mm mesh) and stored at +4 °C for less than 1 month before the experiment. Plant materials were air-dried and mechanically processed into 5–10 mm fragments. Before experimental use, soil samples were moistened to 65% water-holding capacity, while air-dried plant residues were moistened to 400% (as determined in preliminary test).

### 4.3. The Incubation Experiment

**Figure 7:**
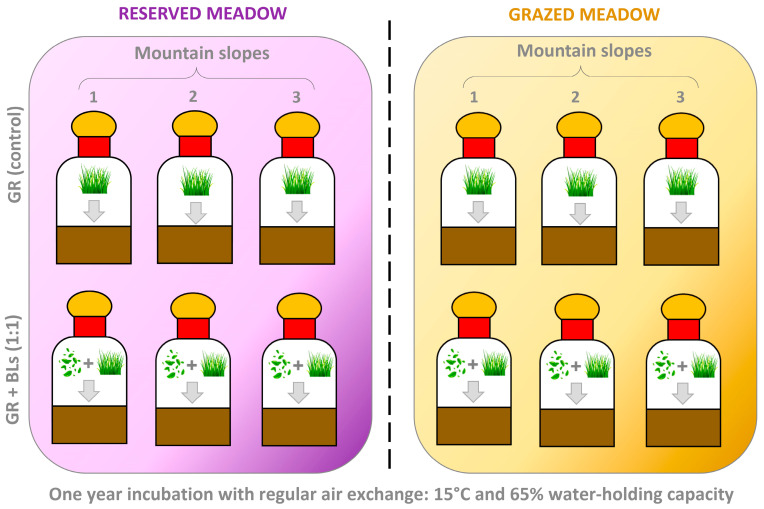
Scheme of the incubation experiment: the addition of the prepared grass residue (GR) as a control and a 1:1 mixture of GR and birch leaves (GR + BLs) to soil sample (300 mL) for each mountain slope in a 1 L vessel.

Prepared soil samples (300 mL) from each mountain slope were placed in 1 L vessels in two variants: (1) addition of prepared GR residues (control) and (2) addition of a 1:1 GR + BLs mixture to simulate treeline encroachment (Figure 7). Plant residues were mixed with soil at a 3:100 ratio (residue–soil; air-dried weight) to simulate plant residue inputs for three growing seasons. This proportion of plant residues was determined based on our previous findings regarding aboveground biomass incorporation in subalpine meadows [38]. The soil–residue mixtures were hermetically sealed in vessels and incubated in the dark at 15 °C for one year, with periodic aeration. The incubation temperature was selected to match growing season conditions, as determined using air temperature data (2020–2021) collected at the study sites using DS1922L-F5 iButton^®^ loggers (Maxim Integrated, San Jose, CA, USA). Initial soil chemical and microbial properties were analyzed prior to residue addition, at the ‘0 moment’ of the experiment. Subsequent analyses were performed after 6 and 12 months of incubation to assess treatment effects on soil properties.

### 4.4. Soil Samples’ Analysis

Total C and N content were analyzed using an ‘ECS 8024-NC’ elemental analyzer (NC Technologies, Bussero, Italy), while total P content was determined using inductively coupled plasma optical emission spectroscopy (ICP-OES 5110; Agilent, Santa Clara, CA, USA). The contents of soil MBC, MBN, and MBP were determined using the fumigation–extraction (FE) method [39,40,41]. For MBC and MBN analysis, soil samples were fumigated with chloroform (24 h in the dark) to destroy microbial cells, followed by extraction with a 0.05 M K_2_SO_4_ solution (1:4 soil/solution ratio). Non-fumigated samples served as controls, providing values for available or dissolved organic carbon (AC) and dissolved total nitrogen (AN). Dissolved C and N concentrations in the extracts from both fumigated and non-fumigated samples were measured using a Topaz NC analyzer (St. Petersburg, Russia). Soil MBC and MBN were calculated as the differences in dissolved C and N between fumigated and non-fumigated samples, divided by conversion factors of 0.45 and 0.54, respectively [39,42]. For MBP and AP determination, soil suspensions were prepared at a 1:10 soil-to-distilled water ratio. An anion-exchange membrane (551642S, VWR International, Darmstadt, Germany, S = 8 cm^2^) and 0.3 mL of chloroform (fumigated sample) or 0.3 mL of distilled water (non-fumigated control) were added to each suspension. The samples were then tightly sealed and incubated in the dark on a rotary shaker (200 rpm) for 24 h. Following this treatment, the membrane was removed from the suspension, washed with distilled water, transferred to 50 mL of 0.25 M H_2_SO_4_ solution, and shaken for 3 h to extract P from the solution. The P content in both fumigated and non-fumigated solutions was determined using an inductively coupled plasma emission spectrometer (Avio 200, No. 68141-17, Singapore, ‘Perkin Elmer Singapore Pte. Ltd.’, 2019). MBP was calculated as the difference between P concentrations in fumigated and non-fumigated samples and adjusted using a soil-specific extraction factor of 0.19 for the studied soils [41]. The P content in non-fumigated samples represents the AP in the soil.

Microbial catabolic activity was assessed using the MicroResp™ system, for non-calcareous soils [43]. Soil samples (~0.3 g each) were placed in 945 μL deep-well plates (96-well format) and amended with aqueous solutions of four groups of organic substrates: carboxylic acids (citric, oxalic), carbohydrates (D-galactose, D-fructose), amino acids (L-arginine, L-aspartic), and phenolic compounds (syringic acid). The 96-deep-well microplate with soil was tightly closed in the 96-well microplate with a detection gel and incubated for 6 h at 25 °C. Absorbance by the detection gel was analyzed at a wavelength of 595 nm (microplate reader FilterMax F5, Molecular Devices, LLC, San Jose, CA USA) before and after incubation, with results expressed as CO_2_ production rates (μg C g^−1^ h^−1^) [44]. Microbial functional diversity was calculated using the Shannon index: H = −ΣP_i_ × lnP_i_, where Pi represents the ratio of the respiration response to substrate i relative to the total respiration response for all studied substrates.

### 4.5. Plant Residuals’ Analysis

The NE (e.g., fats and waxes), WS (e.g., carbohydrates and phenolics), AS (e.g., cellulose), and AIS fractions of the plant materials were determined using a sequential extraction technique [45]. NE was extracted with dichloromethane in a water bath for 24 h. The WS fraction was determined with hot water extraction. Fractions of AS and AIS were divided using lignin content determination. Lignin was isolated by acid hydrolysis in 72% sulfuric acid. This fraction was combusted at 600 °C to determine the ash content. The remaining material was a mineral fraction (MF). The MF fraction was subtracted to calculate the AIS fraction. The content of every fraction was gravimetrically determined (Sartorius CP124S, Goettingen, Germany) and expressed as a percentage of dry weight using extraction coefficients for calculation.

### 4.6. Data Analysis

Significant differences between the two experimental variants during incubation were examined using the nonparametric paired test (Wilcoxon signed-rank test). The relationships between plant residual characteristics and soil microbial properties were analyzed using Pearson’s correlation (n = 12) for each incubation period (6 and 12 months) and visualized using a correlation matrix. For significant pairwise relationships, the coefficient of determination (R^2^) for each incubation period was calculated using simple linear regression analysis. In prior statistical analysis, both dependent and predictor variables were transformed for the 6-month (log10 for MBC and RS_CH_; H^2^ and RS_AA_^4^) and 12-month (H^4^; RS_CX_^2^; RS_PH_^2^) incubation periods according to normal distribution adjustments. Statistical analyses were performed using the RStudio software (version 4.3.2) [46]. The PerformanceAnalytics package was used to visualize the correlation matrix. The line charts and stacked bar chart were generated using Microsoft^®^ Excel^®^ LTSC MSO (16.0.14332.20431).

## 5. Conclusions

Our results confirm Hypothesis 1, demonstrating decreased C, N, and P retention in microbial biomass during early treeline shifts in subalpine meadow soils. The input of leaf litter during treeline advancement increased the proportion of recalcitrant organic compounds, thereby reducing the soil N retention in microbial biomass. Concurrently, treeline shifts are expected to enhance catabolic microbial diversity in both reserved and grazed subalpine soils. This increase in diversity resulted from elevated recalcitrant compounds (i.e., NE and AIS fractions) in plant litter, which balanced the activity between carbohydrate- and carboxylic acid-utilizing microbial groups, confirming Hypothesis 2. Thus, the consequences of the early-stage treeline shift are consistent with our previous findings along the forest–meadow transect, which may be interpreted as a long-term effect [23].

Understanding the contribution of plant residue composition to soil microbial processes related to C, N, and P cycling will allow predictions of the consequences of vegetation change for the best management practices, such as conservation and grazing in subalpine meadows.

## Figures and Tables

**Table 1:** The coefficient of determination (R^2^) indicates the proportion of variation in the dependent variables, namely, microbial characteristics ^1^ explained by the predictor variables—plant residue fractions ^2^—after 6 and 12 months of soil samples incubation. Bold values are significant at ** *p* ≤ 0.05, * *p* ≤ 0.1. The blue and red values show the direct and inverse relationships, respectively.

Fractions	MBC	MBN	MBP	H	RS_CH_	RS_AA_	RS_CX_	RS_PH_
6 months								
WS	0.57 **	0.05	0.15	0.44 **	0.17	0.15	0.31 *	0.35 **
NE	0.01	0.26 *	0.03	0.30 *	0.53 **	0.00	0.61 **	0.24
AS	0.00	0.26 *	0.00	0.51 **	0.56 **	0.00	0.59 **	0.27 *
AIS	0.12	0.43 **	0.02	0.19	0.28 *	0.05	0.28 *	0.06
12 months								
WS	0.57 **	0.16	0.00	0.01	0.01	0.17	0.07	0.07
NE	0.00	0.30 *	0.21	0.11	0.05	0.29 *	0.05	0.05
AS	0.02	0.26 *	0.15	0.02	0.00	0.46 **	0.09	0.00
AIS	0.03	0.14	0.16	0.05	0.00	0.30 *	0.03	0.02
Color scale for R^2^								
0.00	0.15	0.25	0.30	0.35	0.45	0.50	0.60	

Hereafter: ^1^ Microbial biomass carbon (MBC), nitrogen (MBN), and phosphorus (MBP); Shannon diversity index (H); and microbial respiration response to carbohydrates (RS_CH_), amino acids (RS_AA_), carboxylic acids (RS_CX_), and phenolic acid (RS_PH_). ^2^ Non-polar extractive (NE), water-soluble (WS), acid-soluble (AS), and acid-insoluble (AIS) fractions.

**Table 2:** Characteristics of grass cover per 0.5 × 0.5 m plot in subalpine meadows in reserved and grazed slopes (mean ± SE for n = 4). Notes: PC, projective cover; NS, number of species.

Slope	PC (%)	NS	Dominant Species (≥10% of PC)
Reserved			
1	81 ± 20	14 ± 5	*Calamagrostis arundinacea* (Schrad.) DC., *Betonica macrantha* K. Koch, *Trollius ranunculinus* (Sm.) Stearn, *Centaurea phrygia* L., *Carex pallescens* L., *Cirsium simplex* C.A. Mey
2	96 ± 3	9 ± 2	*Calamagrostis arundinacea* (Schrad.) DC., *Betonica macrantha* K. Koch
3	91 ± 10	6 ± 2	*Calamagrostis arundinacea* (Schrad.) DC., *Betonica macrantha* K. Koch
Grazed			
1	46 ± 8	14 ± 4	*Agrostis vinealis* Schreb., *Betonica macrantha* K. Koch
2	66 ± 11	16 ± 1	*Nardus stricta* L., *Potentilla erecta* (L.) Raeusch.
3	97 ± 5	21 ± 2	*Agrostis vinealis* Schreb., *Carum caucasicum* Boiss, *Betonica macrantha* K. Koch, *Avenella flexuosa* (L.) Drejer

## Data Availability

Detailed data of this research are available by contacting the corresponding author.

## Supplementary Materials

The following supporting information can be downloaded at: https://www.mdpi.com/article/10.3390/plants14101541/s1, Figure S1: Correlation matrix for microbial characteristics and plant residue quality after 6 months of soil sample incubation; Figure S2: Correlation matrix for microbial characteristics and plant residue quality after 12 months of soil sample incubation; Table S1: Mean differences among the contents of total C, N, and P and their available forms, as well as microbial biomass C, N, and P in soil samples supplemented with subalpine GR and a mixture of GR + BLs.
