# Supplementary material for: Effects of Early-Stage Treeline Shifts on Soil Microbial Biomass and Catabolic Diversity in Reserved and Grazed Subalpine Meadows
Source: Plants (Basel). 2025 May 20;14(10):1541. doi: 10.3390/plants14101541 (PMC12115023; doi:10.3390/plants14101541)
Supplement: Supplementary file 1 [file plants-14-01541-s001.zip › plants-3595065-supplementary.pdf]

Supplementary materials

Table S1. Mean differences (M.diff) among the contents of total carbon (C), nitrogen (N), and phosphorus (P) and their available forms (AC, AN, and AP, respectively) as well as microbial biomass C, N, and P (MBC, MBN, and MBP, respectively) in soil samples with subalpine grass and the mixture of grass and birch leaves. NS, not significant. Wilcoxon signed-rank test (*n*=3), significance level is 0.05.

Parameter	Reserved slopes		Grazed slopes	
	M.diff 6 months	M.diff 12 months	M.diff 6 months	M.diff 12 months
Total, g kg^{-1}				
C	2.23^{NS}	4.74^{NS}	-0.67^{NS}	8.18^{NS}
N	-0.15^{NS}	0.27^{NS}	-0.31^{NS}	0.47^{NS}
P	0.00^{NS}	-0.09^{NS}	-0.07^{NS}	0.08^{NS}
Available, mg kg^{-1}				
AC	-263^{NS}	44^{NS}	22^{NS}	-38^{NS}
AN	-53^{NS}	-45^{NS}	-114^{NS}	-170^{NS}
AP	-0.95^{NS}	-41^{NS}	-0.05^{NS}	-0.23^{NS}
Microbial, mg kg^{-1}				
MBC	534^{NS}	465^{NS}	84^{NS}	-123^{NS}
MBN	96^{NS}	216^{NS}	172^{NS}	188^{NS}
MBP	-3.5^{NS}	25^{NS}	39^{NS}	34^{NS}

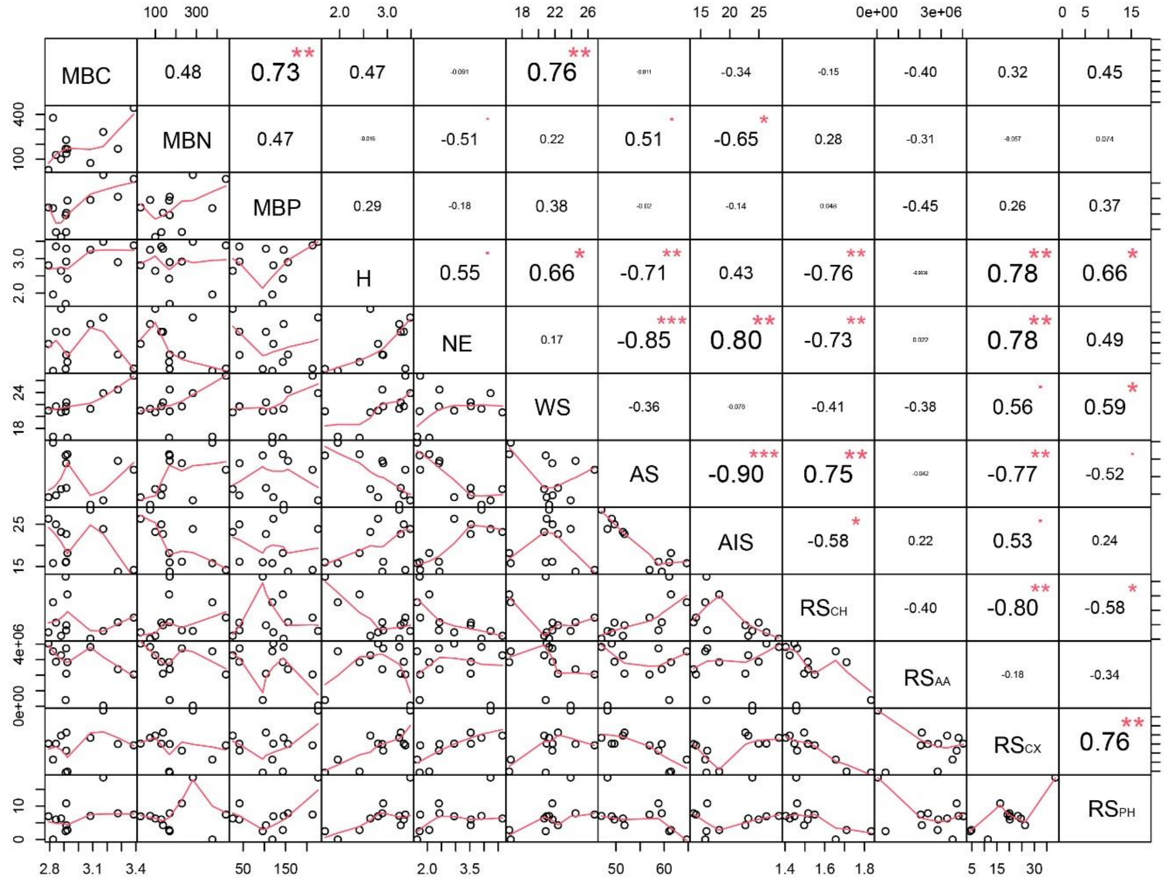

Figure S1. Correlation matrix for microbial characteristics[1] (MBC, MBN, MBP, H, RS$_{CH}$, RS$_{AA}$, RS$_{CX}$, RS$_{PH}$) and plant residue quality[2] (WS, NE, AS, AIS) after 6 months of soil sample incubation. n=12, values are Pearson's correlation coefficients. Significance level: *0.1, **0.05, and ***0.001.

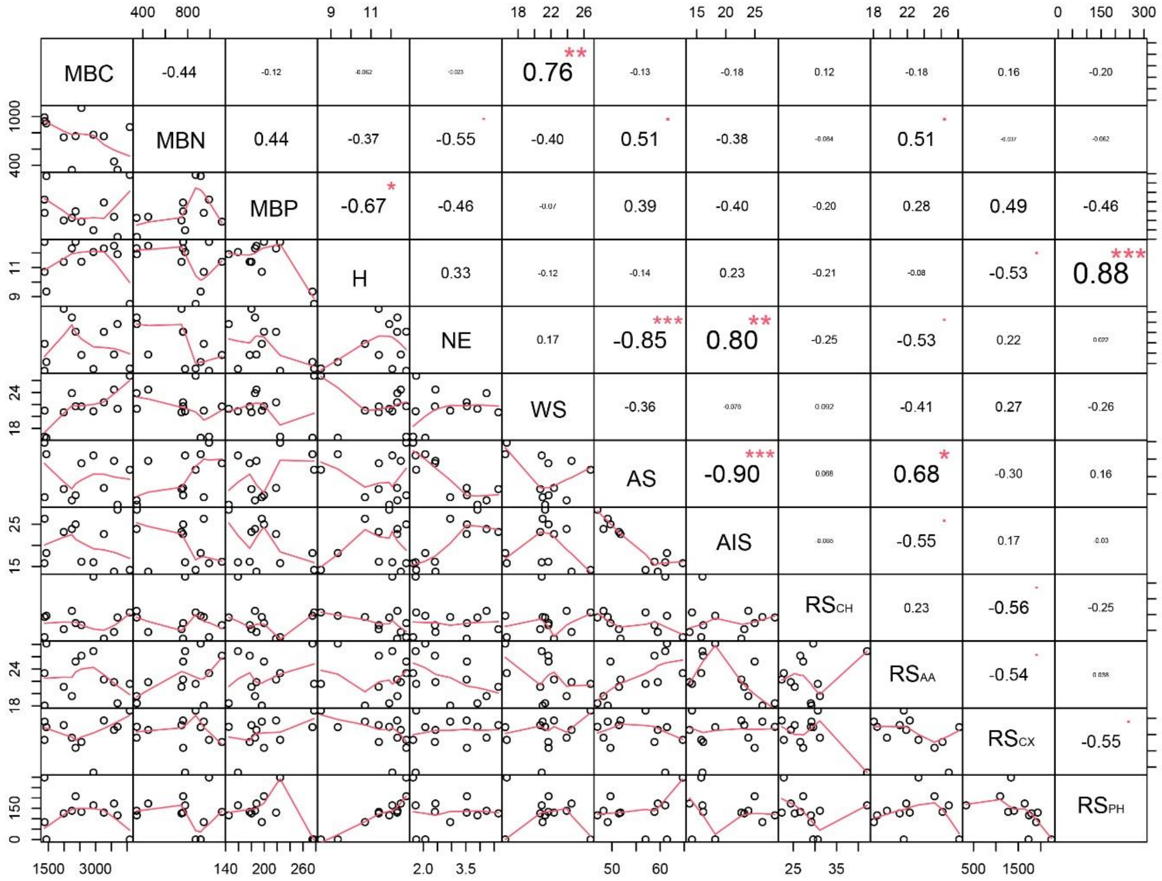

Figure S2. Correlation matrix for microbial characteristics[1] (MBC, MBN, MBP, H, RS_{CH}, RS_{AA}, RS_{CX}, RS_{PH}) and plant residue quality[2] (WS, NE, AS, AIS) after 12 months of soil sample incubation. n=12, values are Pearson's correlation coefficients. Significance level: *0.1, **0.05, and ***0.001.
